# PCB-77 biodegradation potential of biosurfactant producing bacterial isolates recovered from contaminated soil

**DOI:** 10.3389/fmicb.2022.952374

**Published:** 2022-09-26

**Authors:** Monika Sandhu, Atish T. Paul, Jarosław Proćków, José Manuel Pérez de la Lastra, Prabhat N. Jha

**Affiliations:** ^1^Department of Biological Sciences, Birla Institute of Technology and Science Pilani, Pilani, Rajasthan, India; ^2^Department of Pharmacy, Birla Institute of Technology and Science Pilani, Pilani, Rajasthan, India; ^3^Department of Plant Biology, Institute of Environmental Biology, Wrocław University of Environmental and Life Sciences, Wrocław, Poland; ^4^Biotecnología de Macromoléculas, Instituto de Productos Naturales y Agrobiología (IPNA-CSIC), San Cristóbal de la Laguna, Spain

**Keywords:** polychlorinated biphenyl, biodegradation, biosurfactant, *Pseudomonas aeruginosa*, *Pseudomonas plecoglossicida*, *Priestia megaterium*, *Brucella anthropi*

## Abstract

Polychlorinated biphenyls (PCBs) are persistent organic pollutants widely distributed in the environment and possess deleterious health effects. The main objective of the study was to obtain bacterial isolates from PCB-contaminated soil for enhanced biodegradation of PCB-77. Selective enrichment resulted in the isolation of 33 strains of PCB-contaminated soil nearby Bhilai steel plant, Chhattisgarh, India. Based on the prominent growth using biphenyl as the sole carbon source and the confirmation of its degradation by GC-MS/MS analysis, four isolates were selected for further study. The isolates identified by 16S rRNA gene sequencing were *Pseudomonas aeruginosa* MAPB-2, *Pseudomonas plecoglossicida* MAPB-6, *Brucella anthropi* MAPB-9, and *Priestia megaterium* MAPB-27. The isolate MAPB-9 showed a degradation of 66.15% biphenyl, while MAPB-2, MAPB-6, and MAPB-27 showed a degradation of 62.06, 57.02, and 56.55%, respectively in 48 h. Additionally, the degradation ability of these strains was enhanced with addition of co-metabolite glucose (0.2%) in the culture medium. Addition of glucose showed 100% degradation of biphenyl by MAPB-9, in 48 h, while MAPB-6, MAPB-2, and MAPB-27 showed 97.1, 67.5, and 53.3% degradation, respectively as analyzed by GC-MS/MS. Furthermore, in the presence of inducer, PCB-77 was found to be 59.89, 30.49, 27.19, and 4.43% degraded by MAPB-6, MAPB-9, MAPB-2, and MAPB-27, respectively in 7 d. The production of biosurfactants that aid in biodegradation process were observed in all the isolates. This was confirmed by ATR-FTIR analysis that showed the presence of major functional groups (CH_2_, CH_3_, CH, = CH_2_, C–O–C, C-O) of the biosurfactant. The biosurfactants were further identified by HPTLC and GC-MS/MS analysis. Present study is the first to report PCB-77 degradation potential of *Pseudomonas aeruginosa, B. anthropi, Pseudomonas plecoglossicida*, and *Priestia megaterium*. Similarly, this is the first report on *Pseudomonas plecoglossicida* and *Priestia megaterium* for PCB biodegradation. Our results suggest that the above isolates can be used for the biodegradation of biphenyl and PCB-77 in PCB-contaminated soil.

## Introduction

Polychlorinated biphenyls (PCBs), belong to a broad family of anthropogenic organic chemicals and are among the group of harmful persistent organic pollutants (POPs) listed under the Stockholm Convention (Zhu et al., 2022). Due to their physical and chemical properties, they are toxic, and bioaccumulate through the food chain that in turn poses a risk to human health and the environment (Pieper, 2005; Tomza-Marciniak et al., 2019). Bioremediation is considered the most promising approach for PCB decontamination from the environment due to its efficiency and cost-effectiveness (Tandlich et al., 2011). Bacteria play a significant role in the remediation of the PCB-contaminated environment (Jing et al., 2018). Many PCB-degrading bacteria have been isolated including Gram-negative strains belonging to the genera *Achromobacter* (Hong et al., 2009), *Burkholderia* (Totevova et al., 2002; Goris et al., 2004), *Pseudomonas* (Hatamian-Zarmi et al., 2009; Kimura et al., 2018; Hirose et al., 2019; Thorat et al., 2020), *Ochrobactrum* (Murinova and Dercov, 2014) and Gram-positive strains belonging to the genera *Rhodococcus* (Seto et al., 1995) and *Bacillus* (Shimura et al., 1999). These bacteria carry out biodegradation (through the metabolic pathway) using enzymes that convert organic pollutants into simpler compounds (Abramowicz, 1990). Biodegradation can be either through mineralization (where an organic pollutant acts as sole source of carbon) or by co-metabolism (where an inducer is required as the second carbon source, transforming the target pollutant at the same time). There are several reports on the use of inducers for effective PCB degradation by bacterial strains belonging to the genera *Acinetobacter, Pseudomonas*, and *Rhodococcus* (Field and Sierra-Alvarez, 2008). The most common inducer that has been used to improve PCB biodegradation is biphenyl, the basic scaffold of all PCB congeners (Luo et al., 2007, 2008). The use of glucose in combination with biphenyl has also been reported to result in improved cell survival (Zoradova-Murinova et al., 2012), and increased biomass (Murinova et al., 2014), and, in turn, enhanced PCB biodegradation. In addition to the use of inducer, bioremediation has been found to be more effective when bacterial growth conditions such as pH, temperature, concentration of the pollutant utilized (Parnell et al., 2010) and production of biosurfactant are optimal. Bioavailability is well known to be one of the most crucial limiting factors for PCB bioremediation (Maier, 2000). Biosurfactants have been reported to improve the bioavailability of PCB to bacterial cells by detaching the strongly adsorbed PCB from the soil (Sifour et al., 2007). Thus, the biosurfactant-producing property of the bacterial strains plays a relevant role in the solubilization of the PCB and in turn makes it bioavailable to bacteria for efficient degradation.

An additional important factor in the biodegradation process is the number and position of the chlorine atom attached to the biphenyl ring. It is reported that the rate of PCB degradation decreases with increasing chlorine content, indicating that highly chlorinated congeners are difficult to degrade (Kohler et al., 1988; Passatore et al., 2014). PCBs with one or two chlorines have been reported to biodegrade mainly at the *para* position *via* mineralization or co-metabolization. For instance, *Achromobacter xylosoxidans* IR08 degraded 4,4′-dichlorobiphenyl (diCB) metabolically (Ilori et al., 2008), while *Rhodococcus* sp. MAPB-1 (Sandhu et al., 2020) and *Rhodococcus* sp. WAY2 (Garrido-Sanz et al., 2020) degraded it cometabolically. Similarly, it has been observed that bacteria exhibit no or reduced degrading ability for PCBs with four or more chlorine substitutions. For example, *Rhodococcus ruber* SS1 and *Rhodococcus pyridinivorans* SS2 efficiently degraded CB, diCB, and triCB, but could not degrade tetraCB and hexaCB (Wang et al., 2016).

PCBs in commercial and environmental samples are complex mixtures of congeners. PCB-77 (3,3′,4,4′-tetrachlorobiphenyl) is one of the major components of commercially available PCB blends and products. It is one of the most toxic coplanar congeners and is ubiquitous in the environment (Meggo et al., 2013). Structure-toxicity studies have revealed that it binds to Aryl hydrocarbon receptor agonists (Ah) that contribute significantly to its toxicity (Safe, 1994). Moreover, its dioxin-like structure makes it highly resistant to biodegradation and there are very few reports on its bioremediations (Liang et al., 2014). Therefore, the objective of the current research was to investigate and optimize the growth of PCB degraders co-metabolically in glucose and biphenyl and to explore their PCB-77 biodegradation abilities. *Pseudomonas aeruginosa* and *Ochrobactrum anthropi* have been previously reported to utilize lower chlorinated biphenyls such as the diCB as the sole source of carbon, while *Pseudomonas plecoglossicida* and *Priestia megaterium* have been reported for the first time for their PCB degradation potential. Furthermore, this is the first report of these bacterial isolates using and degrading PCB-77.

## Materials and methods

### Chemicals and media

LB medium, minimal medium (MM), and glucose were purchased from Himedia (Mumbai, India). Cetyltrimethylammonium bromide (CTAB), methylene blue, biphenyl, PCB-77 (3,4,3′,4′-tetrachlorobiphenyl), acetone, ethyl acetate, hexane, methanol (Empure, GC grade) was purchased from Sigma Aldrich (St. Louis, MO, USA).

#### Selective enrichment and isolation of microorganisms from polychlorinated biphenyl contaminated soil

The soil samples were collected at a depth of 0–10 cm from the nearby site of Bhilai Steel Plant, Chhattisgarh, India, at latitude 21.1915°N and longitude 81.4041°E. Selective enrichments were made by inoculating 50 ml sterile MM with 10 g of PCB-contaminated soil samples. In our previous studies, GC-MS/MS analysis showed that the soil samples collected were contaminated with PCB (Sandhu et al., 2022). MM was supplemented with 1% biphenyl (*w/v*) as a sole carbon source and was incubated on a rotary shaker (150 rpm) at 30°C. After 1 week of the acclimatization period, 1% of the inoculum was then transferred to fresh 50 ml of MM supplemented with 1% biphenyl, repeating up to three generations at a 7-day interval. Serial dilution was done from enriched diluent up to 10^–6^ and 100 μl from each sample was plated onto MM Agar medium supplemented with 1% (*w/v*) biphenyl as a carbon source. The plates were incubated at 30°C to isolate PCB-degrading bacteria. The bacterial colonies of different morphotypes were further grown in MM and were screened based on their growth measured by optical density (OD) at 600 nm for further PCB degradation experiments. Four selected bacterial isolates were further observed for different morphological characteristics such as shape, color, and colony pattern. These isolates were subjected to biochemical tests such as Gram staining, carbohydrate utilization, catalase, oxidase, starch hydrolysis, indole, methyl red, and Voges-Proskauer tests. These tests were performed using biochemical test kits (HiMedia, Mumbai, India) according to the manufacturer’s instructions.

### Identification of bacterial isolates and PCR amplification of dioxygenase gene

The bacterial isolates were inoculated in 5 ml LB tubes and grown at 30°C for 16 h with150 rpm shaking condition. Bacterial genomic DNA was isolated using the QIAamp kit (Qiagen, USA) and detected by agarose gel electrophoresis (1%; *w/v*). The DNA template (50 ng) of each isolate was used for the amplification of the 16S rRNA gene using universal primers 27F (5′-AGAGTTTGATCCTGGCTCAG-3′) and 1494R (5′-CTACGGCTACCTTGTTACGA-3′) with a thermal cycler (T100, BioRad, USA). The temperature conditions were set as follows: initial denaturation of 5 min at 94°C, 30 cycles of 1 min at 95°C, 1 min at 53° and extension of 2 min at 72°C, and final extension of 5 min at 72°C. The amplicon was purified and sequenced using the dideoxy chain terminator method in the DNA sequencing facility of the Department of Biochemistry, South Campus, Delhi University (New Delhi, India). 16S rRNA sequence was compared with the National Centre for Biotechnology Information (NCBI) public database using the BLAST tool^1^ and was deposited in the NCBI/Gen Bank nucleotide sequence database (refer to accession number in Table 1). The sequence was further aligned with the reference sequence using MEGA XI (Tamura et al., 2021) for the construction of the phylogenetic tree.

**TABLE 1:** Thirty-three bacterial strains isolated from PCB contaminated soil and sequence deposited with provided accession no. in NCBI database.

Isolates	Percent identity	Percent coverage	Closest type strain	NCBI accession no.
MAPB1	99.72	100	*Pseudomonas plecoglossicida* NBRC 103162^T^	MK512369.1
MAPB2	100	100	*Pseudomonas aeruginosa* JCM5962^T^	MK512370.1
MAPB3	97.93	100	*Ochrobactrum anthropi* ATCC 49188^T^	MK512371.1
MAPB4	100	100	*Pseudomonas plecoglossicida* DSM 15088^T^	MK512372.1
MAPB5	100	100	*Pseudomonas monteilii* CIP 104883^T^; AF064458^T^	MK512373.1
MAPB6	99.30	100	*Pseudomonas plecoglossicida* FPC951^T^	MK512374.1
MAPB7	99.43	99	*Brevibacterium iodinum* DSM 20626^T^	MK512375.1
MAPB8	99.57	96	*Pantoea dispersa* LMG2603^T^; DQ504305^T^	MK512376.1
MAPB9	100	100	*Brucella anthropi* ATCC 49188^T^	MK512377.1
MAPB10	99	100	*Bacillus amyloliquefaciens* NBRC 15535^T^	MW227569.1
MAPB11	99.79	99	*Bacillus stratosphericus* 41KF2a^T^; AJ831841^T^	MW227570.1
MAPB12	98.75	100	*Lysinibacillus composti* NCCP-36^T^; AB547124^T^	MW227571.1
MAPB13	99.50	100	*Bacillus cereus* ATCC 14579^T^; AE016877^T^	MW227572.1
MAPB14	100	100	*Bacillus aryabhattai* B8W22^T^	MW227573.1
MAPB15	98.34	99	*Solibacillus silvestris* HR3-23^T^; AJ006086^T^	MW227574.1
MAPB16	100	100	*Bacillus thuringiensis* IAM 12077^T^; ATCC 10792^T^	MW227575.1
MAPB17	100	100	*Bacillus cereus* ATCC 14579^T^; AE016877^T^	MW227576.1
MAPB18	100	100	*Bacillus paramycoides* MCCC 1A04098^T^	MW227577.1
MAPB19	99.79	100	*Bacillus aryabhattai* B8W22^T^; EF114313^T^	MW227578.1
MAPB20	98.54	100	*Bordetella petrii* DSM 12804^T^; AJ249861^T^	MW227579.1
MAPB21	99.79	100	*Staphylococcus pasteuri* ATCC 51129^T^	MW227580.1
MAPB22	98.96	99	*Pseudomonas aeruginosa* JCM 5962^T^; DSM50071^T^	MW227581.1
MAPB23	99	100	*Bacillus cereus* ATCC 14579^T^; AE016877^T^	MW227582.1
MAPB24	99.79	99	*Paenibacillus illinoisensis* JCM 9907^T^; AB073192^T^	MW227583.1
MAPB25	99.38	100	*Bacillus amyloliquefaciens* NBRC 15535^T^	MW227584.1
MAPB26	98	100	*Bacillus velezensis* strain CR-502^T^	MW227585.1
MAPB27	100	100	*Bacillus megaterium* NBRC 15308^T^; IAM 13418^T^	MW227586.1
MAPB28	99.79	100	*Bacillus aryabhattai* B8W22^T^; EF114313^T^	MW227587.1
MAPB29	100	100	*Bacillus subtilis* DSM10^T^; AJ276351^T^	MW227588.1
MAPB30	99.79	100	*Bacillus subtilis* DSM10^T^; AJ276351^T^	MW227589.1
MAPB31	92.48	99	*Domibacillus indicus* strain SD111	MW227590.1
MAPB32	100	99	*Bacillus cereus* ATCC 14579^T^; AE016877^T^	MW227591.1
MAPB33	99.79	99	*Paenibacillus illinoisensis* JCM 9907^T^; AB07319^T^	MW227592.1

In the initial step of the biphenyl catabolic pathway, biphenyl is converted into 2,3-dihydroxy-1-phenylcyclohexa-4,6-diene (dihydrodiol compound) by an aromatic-ring-hydroxylating dioxygenase (ARHD), biphenyl 2,3-dioxygenase (BDO), which consists of the large and small subunits of terminal oxygenase, ferredoxin, and ferredoxin reductase. The PCR reaction was also performed to amplify the aromatic ring hydroxylating dioxygenase (ARHD) gene in the selected bacterial isolate MAPN-2, MAPB-6, MAPB-9, and MAPB-27 using specific primers. Primers were designed using Primer3 software and the details are provided in Table 2. PCR amplification was carried out in a reaction volume of 25 μl containing 2.5 μl Taq DNA polymerase buffer (10X), 0.35 μl of Taq DNA polymerase (3U), 0.3 μl dNTP (2.5 mM each), 1.0 μl of primer (10 mM each) and 3 μl (50 ng) of the template. Thermocycler T100 (BioRad, Germany) was set for an initial denaturation step of 4 min at 95°C, followed by 25 cycles of denaturation at 94°C for 30 s, annealing at the respective temperature for each pair of primers for 45 s (Table 2), extension at 72°C for 30 s and a final extension step at 72°C for 5 min. The PCR amplicon was analyzed on a 1.5% agarose gel under a UV gel documentation unit (Biorad, USA).

**TABLE 2:** Primer used in this study.

Isolates	Primer	Primer sequence	Annealing temp.(°C)	Amplicon size (bp)
*Universal 16S*	27 F 1494 R	5′-AGAGTTTGATCCTGGCTCAG-3′ 5′-CTACGGCTACCTTGTTACGA-3′	53	1500
*Pseudomonas aeruginosa* MAPB-2	PA-ARHD F PA-ARHD R	5′-GGCCAGGCGAAGGACTATAT-3′ 5′-GTGCCGAGGGTATTCAGGTA-3′	57	157
*Pseudomonas plecoglossicida* MAPB-6	PP-ARHD F PP-ARHD R	5′-AGAAGCTTTTACCCTGCCCT-3′ 5′-GGAACGCATGAATCTGTCCC-3′	57	199
*Brucella anthropi* MAPB-9	BA-ARH F BA-ARH R	5′-GACCAGCTGGAGAAGCAGAT-3′ 5′-TGAACCCCTTCGACAGATTC-3′	57	161
*Priestia megaterium* MAPB-27	BM-ARHD F BM-ARHD R	5′-ACCGCACGTATTTTGGCATT-3′ 5′-CACCACTCACACGCTTCAAA-3′	57	202

### Optimization of growth parameter for potential polychlorinated biphenyl-degrading bacterial isolates

Stock solution (1 mg/ml) of biphenyl was prepared in acetone. Different concentrations were further prepared in sterile flasks by using the stock solution. Biodegradation experiments were carried out in two different parallel sets. The first set consisted of isolates grown in the presence of various concentrations of biphenyl (i.e., 10, 50, 100, 200, 300, 400, and 500 mg/l) for 48 h. Additionally, the biphenyl concentration that shows the maximum growth for all the selected isolates was used for the second experimental set with different glucose concentrations (0.1, 0.2, 1, 2, and 3%, *w/v*). Glucose is the carbon source that bacteria most easily utilize and therefore was used to promote growth in biphenyl-supplied media and to enhance degradation. The concentration of biphenyl and glucose at which maximum bacterial growth was obtained from the previous experiment was further used to optimize the growth conditions at temperature (20 to 40°C with 5°C intervals) and pH (4–9). The growth of MAPB-2, MAPB-6, MAPB-9, and MAPB-27 was monitored by measuring the OD_600_ after 48 h of the incubation period1% (*w/v*). The plates were incubated at 30°C for 48 h with subsequent counting of colonies. All experiments were performed in triplicate.

### RNA extraction and reverse transcription polymerase chain reaction

Selected bacterial strains MAPB-2, MAPB-6, MAPB-9, and MAPB-27 were inoculated in MM supplemented with different concentration (100–300 mg/l) of biphenyl and grown for 72 h at standard conditions. After the incubation period, bacterial culture was collected by centrifugation at 10,000 × *g* at 4°C for 8 min. Total RNA was extracted as per the modified Trizol method (Rio et al., 2010) and was stored at −80°C. The integrity of RNA was verified by 1% agarose gel electrophoresis, and the determination of RNA concentration and purity were checked by Nano-drop spectrophotometer. 20 μL of the reaction mix containing 1 μL Verso enzyme mix, 2 μL dNTP mix, 1 μL RNA primer, 1 μL RT enhancer, 4 μL 5 × cDNA synthesis buffer, 1 μg template RNA and RNasefree water was used for reverse transcription. The reaction mix was incubated at 42°C for 30 min and, then the enzyme was inactivated at 95°C for 2 s. The cDNA was stored at −20°C. A set of 16S rRNA primer and genus-specific primers targeting the aromatic ring hydroxylating dioxygenase gene (ARHD) of degradation pathway were used for RT-qPCR assays. Melting curve and efficiency of the selected primer set were evaluated by RT-qPCR using CFX96 Real time detection system (BioRad, USA). DNA (2 μL) from the experimental group and the control group was used as a template for RT-qPCR. The reaction mixture contained 2X Power SYBR Green Master mix (Applied Biosystems™), 1 μL of forward and reverse primer, 1 μL of cDNA template and nuclease free water to make up to 10 μL. The cycling conditions consisted of an initial step of 5 min at 95°C, followed by 40 cycles of denaturation at 94°C for 30 s, annealing at 57°C for 45 s and elongation at 72°C for 5 min. RT-qPCR triplicates of each biological replicates were performed. The relative expression level was determined as the fold change in accordance with the 2^–△ △^ *^Ct^* method (Livak and Schmittgen, 2001).

### Biodegradation assay with biphenyl and PCB-77

#### Biodegradation assay with biphenyl

In order to investigate the difference in the biphenyl degradation by the selected isolates, two different sets of experiments were carried out. The first set consisted of 200 mg/l biphenyl as the sole carbon source in 20 ml MM inoculated with 10% inoculum. The tubes were cultured at 30°C, 150 rpm for 48 h of incubation. The second set consisted of 0.2% glucose as a co-substrate along with 200 mg/l biphenyl with optimized growth parameters.

#### Biodegradation assay with PCB-77

For PCB degradation assay, PCB-77 congener was used as it is one of the most toxic coplanar congeners and is widely used in commercially available blends such as Aroclor (Venkatachalam et al., 2008). For the PCB-77 assay, the inoculating medium consist of MM supplemented with 50 mg/l biphenyl, dissolved in acetone. Media was inoculated with selected isolates and incubated at 30°C for 48 h. After the incubation period, inoculation media was centrifuged and the pellet was washed with phosphate buffer (pH 7.4). The bacterial pellet was resuspended with MM and 10% of the culture inoculum was added to assay media containing MM supplemented with PCB-77 (50 mg/l). The flasks were incubated for 7 days at 30°C, 150 rpm.

For both assays, after the incubation period, the extraction of the sample was done to determine the percentage of biodegradation. The sample was extracted three times with hexane. The hexane layer was collected and evaporated by a rotary evaporator. The concentrated extracts were resuspended with isooctane. 1 μl extract of each sample was used for GC-MS/MS analysis, using a GC-MS/MS TQ8040 (Shimadzu, Japan). The separation of biphenyl and PCB-77 was performed on an SH-RXi-5SilMS fused silica column with dimensions of 30 m × 0.32 mm × 0.25 μm. The column temperature was programmed from 80°C (1 min hold) to 150°C at 25°C/min and finally to 280°C at 4°C/min (2 min hold) as per modified method of Wang et al. (2016). The carrier gas was helium and the column flow rate was 1ml/min. The injector, ion source, and detector temperature were 250, 250, and 270°C, respectively. The areas of specific peaks in the chromatogram of the samples were measured and compared to those of control samples supplemented with PCB-77 (50 mg/l) without inoculum. The residual biphenyl/PCB-77 was estimated by plotting the calibration curve of biphenyl/PCB-77 at different concentrations. Biphenyl calibration curve was prepared using different concentrations of standard solutions (50, 100, 150, and 200 mg/l; *y* = 35124x+900526, *R*^2^ = 0.9897). The recoveries of biphenyl from the spiked samples were in the range of 96.98–99.10%. The calibration curve of PCB-77 was constructed with different concentrations of 50, 100, 150, and 200 mg/l (*y* = 28025x-617466, *R*^2^ = 0.9871). The recoveries of PCB-77 from the standards were in the range of 90–96%. The percentage of biphenyl/PCB-77 degradation was calculated as follows.


(1) $$Percentdegradation(\%)=\frac{Peakareaofcontrol-Peakareaofbiphenyl/PCB-77}{PeakareaofControl}\times 100$$

##### Analysis of biphenyl and PCB-77 metabolites

For the identification of metabolites produced during biphenyl degradation, the obtained extracts were derivatized with 100 μl of bis (trimethylsilyl) trifluoroacetamide (BSTFA) and trimethylchlorosilane (TMCS); (99:1, *v/v*) at 60°C for 15 min (He et al., 2015) and were analyzed by GC-MS/MS as described above.

### Production and characterization of biosurfactant

#### Preliminary test for the detection of biosurfactant production

The biosurfactant production of each isolate was tested using a semi-quantitative CTAB agar assay according to the method of Perfumo et al. (2006). CTAB agar plates (per liter) were prepared by adding 0.2 g of CTAB, 0.005 g of methylene blue to MM containing 20 g of glucose, 1 g of yeast extract, 2 g of peptone, 0.1 g of CaCl_2_, and 15 g of agar. The plates were inoculated with 20 μl culture and incubated at 30°C for 48 h. The production of biosurfactants as extracellular glycolipids by bacterial isolates could be confirmed by the formation of dark blue halos around the colonies.

#### Production and characterization of biosurfactants

For the production and characterization of biosurfactants, Bushnell-Haas broth (BH) was supplemented with 1.5 g/l NaNO_3_ and 2% glycerol. The medium was inoculated with isolates and kept for 7 d at 30°C, 150 rpm for incubation. After the incubation period, the suspension was centrifuged at 6400 g for 30 min. The supernatant was collected and acidified to pH 2 with HCl (6N). The acidified supernatant was kept overnight at 4°C to precipitate the biosurfactant. An equal volume of chloroform and methanol (2:1; *v/v*) was added to the supernatant and vigorously shaken for 10 min to extract the biosurfactant produced. The organic layer was collected and evaporated by a rotary evaporator (Tripathi et al., 2020). The supernatant was tested for its emulsifying activity and drop collapse assay, while a crude biosurfactant was used for further characterization using ATR- FTIR (Attenuated total reflectance - Fourier transform infrared spectroscopy), HPTLC, and GC-MS/MS techniques.

#### Emulsification and drop collapse assay

Further characterization of the biosurfactant was performed by calculating the emulsification capacity. It was carried out by mixing petrol and culture supernatant (1:1 *%*) in a test tube. The solution was vortexed for 1 min and kept at room temperature for 24 h. The *E*_24_ emulsification index was calculated for the selected isolates as indicated below.


$$Emulsification\;indexE24(\%)=\frac{heightoftheemulsionlayer\left(cm\right)}{Totalheightofthesolution\left(cm\right)}\times 100$$

A qualitative drop collapse test was performed by adding 10 μl of petroleum and 10 μl of supernatant to the surface of the oil. The shape of the drop on the oil surface was observed after 3 min. The culture supernatant that makes the oil drop collapse was indicated as a positive score and the drops that remained beaded indicated as a negative score. Distilled water was used as negative control (Jimoh and Lin, 2019).

#### HPTLC and ATR-FTIR characterization of biosurfactant

To determine the type of biosurfactant, 10 μl of the crude extract was separated on silica gel TLC plates (F_254_; Merck, Germany) by using HPTLC (Camag, Switzerland) with Software visionCATS. The solvent system used was a mixture of chloroform, methanol, and acetic acid in the ratio of 6.5:1.5:0.2 (*v/v/v*). The bands were visualized by a TLC scanner under 254 nm, 366 nm, and white light after anisaldehyde derivatization for the detection of carbohydrates. The plates were air-dried, dipped in anisaldehyde reagent acetic acid: sulphuric acid: p-anisaldehyde (100: 2: 1; *v/v/v*), and heated for 15 min at 110°C. The retention factor (R_*f*_ value) of each band was determined.

Characterization of functional groups in the biosurfactant produced by the selected isolates was done by ATR-FTIR (Bruker, Germany). Analysis of the IR spectra at the mid-infra-red region of 400–4,000 cm^–1^ was carried out by OPUS software 7.0 in transmittance mode.

#### GC-MS/MS analysis for identification of biosurfactant composition

GC-MS/MS analysis was performed for revealing the fatty acid composition of the produced biosurfactant. Extract (1 μl) was injected into the RTX-5Sil column with initial temperature maintained at 50°C for 2 min, then programmed to 80°C at a rate of 10°C/min (5 min). The rate was increased further by 5°C/min up to 100°C with a hold of 1 min and the final increase with 15°C/min to 280°C with a hold of 5 min. The flow rate was 1 ml/min. The injection and detector temperatures were set at 200°C and 230°C, respectively. The MS scan range was kept at 45–500 *m/z*.

### Statistical analysis

All the experiments were done in triplicates and data is represented in ± standard error mean.

## Results and discussion

### Selective enrichments for the isolation and identification of polychlorinated biphenyl-degrading bacteria

**FIGURE 1:**
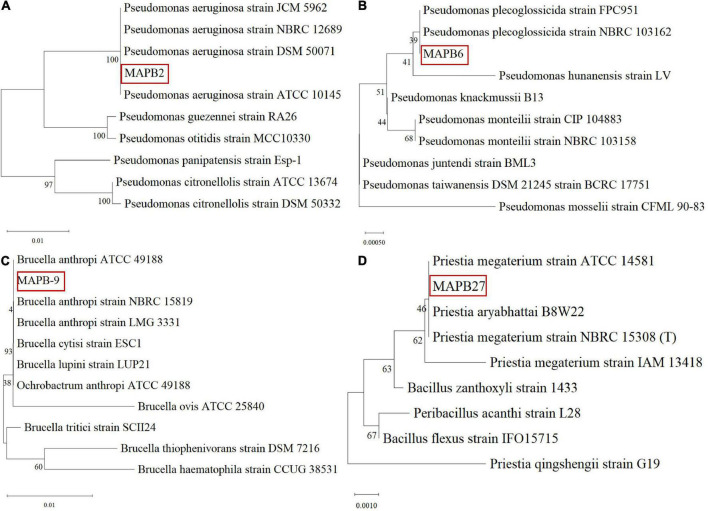
Phylogenetic tree by Maximum Likelihood method and Kimura 2-parameter model based on 16S rRNA gene sequences **(A)** MAPB-2, **(B)** MAPB-6, **(C)** MAPB-9, and **(D)** MAPB-27. Bootstrap values (expressed as percentages generated from 1,000 replicates) are shown at branch points.

Repetitive enrichment and subsequent subculturing of soil in MM with biphenyl at 30°C for 7 days resulted in 33 morphologically distinct bacteria. All 33 bacterial isolates were identified by 16S rRNA gene sequencing analysis and the nucleotides were deposited in the NCBI GenBank database (Table 1). Most of the isolated bacteria belong to phylum Firmicutes followed by Proteobacteria and Actinobacteria. The abundance of these phyla in PCB contaminated soil is in accordance with the other reported studies such as the predominance of Firmicutes and Proteobacteria in aged soil contaminated with PCB (Cervantes-González et al., 2019); and the enrichment of Proteobacteria and Acidobacteria in the presence of PCB congeners and Aroclor 1224 (Correa et al., 2010). Each isolate was separately screened to assess its ability to biodegrade biphenyl as a sole carbon and energy source. After 7 days, based on prominent growth, as indicated by optical density (OD) in biphenyl, four bacterial isolates, namely MAPB-2, MAPB-6, MAPB-9, and MAPB-27, were selected for further biodegradation studies. The strains MAPB-2, MAPB-6, MAPB-9, and MAPB-27 were identified as organisms belonging to *Pseudomonas aeruginosa, Pseudomonas plecoglossicida, Brucella anthropi*, and *Priesta megaterium*, respectively. Furthermore, to determine the taxonomic position of each strain, the 16S rRNA gene sequences were aligned with the reference sequences available in the NCBI database. The phylogenetic tree was constructed using the Maximum likelihood method and Kimura 2-parameter model (Kimura, 1980) with a bootstrap test of 1,000 replicates (Figure 1) with Mega XI (Tamura et al., 2021). Initial tree(s) for the heuristic search were obtained automatically by applying Neighbor-Joining and BioNJ algorithms to a matrix of pairwise distances that were estimated using the Maximum Composite Likelihood (MCL) approach, and then selecting the topology with a superior log likelihood value. Investigation of the 16S rRNA gene tree showed that strain MAPB-2 is a member of the genus *Pseudomonas*, forming a cluster with *Pseudomonas aeruginosa* DSM50071*^T^* and *Pseudomonas aeruginosa* ATCC 10145*^T^* (Figure 1A), the strain MAPB-6 is a member of the genus *Pseudomonas*, forming a cluster with *Pseudomonas plecoglossicida* NBRC 103162*^T^* and *Pseudomonas plecoglossicida* FPC951*^T^* (Figure 1B)*;* the strain MAPB-9 is a member of the genus *Brucella*, forming a cluster with *Brucella anthropi* ATCC 49188*^T^* and *Brucella anthropi* NBRC 15819^ T^ (Figure 1C); while the strain MAPB-27 is a member of the genus *Priestia*, forming a cluster with *Priestia megaterium* ATCC 14581*^T^* and *Priestia aryabhattai* B8W22*^T^* (Figure 1D). *Ochrobactrum* and *Bacillus* has been reclassified into genus *Brucella* (Hordt et al., 2020) and *Priestia* (Gupta et al., 2020), respectively. The degradation of PCBs by various species of genera *Pseudomonas, Ochrobactrum*, and *Bacillus* such as *Pseudomonas* sp. 2 (Komancova et al., 2003), *Ochrobactrum* sp. NP04 (Pathiraja et al., 2019), *Ochrobactrum intermedium* (Liz et al., 2009), *B. anthropi* (Horvathova et al., 2018), and *Bacillus* sp. have been reported in earlier studies. However, this is the first report of *Pseudomonas plecoglossicida* and *Priestia megaterium* as potential PCB degraders. These two species were known to degrade organophosphate pesticides (Bhadbhade et al., 2002; Borji et al., 2014). The morphological and biochemical properties of the four selected bacterial isolates were carried out and details are highlighted in Table 3. Based on morphology, the MAPB-2, MAPB-6, MAPB-9, and MAPB-27 isolates were found to be differential rod-shaped with blue-green, green, white, and white colonies on LB agar plates, respectively. MAPB-2, 6, and 8 were Gram-negative, whereas MAPB-27 was found to be Gram-positive. All the isolates grew aerobically and showed positive results for catalase, whereas negative results for Voges Proskauer, methyl red, and indole test. MAPB-2, MAPB-6 and MAPB-9 showed a negative response to O-nitrophenyl-β-D-galactopyranoside (ONPG) and H_2_S production, while MAPB-27 showed a positive response. Different carbon utilization tests showed that isolates differed in their use of carbon sources as provided in Table 3.

**TABLE 3:** Morphological and biochemical properties of the screened PCB degrader’s.

Screened bacterial isolates	MAPB-2	MAPB-6	MAPB-9	MAPB-27
**Morphological properties**				
Shape	Rods	Rods	Short rods	Long rods
Gram strain	−	−	−	+
**Color**	Bluish green	Green	Pale white	White
**Biochemical test**				
ONPG	−	−	−	+
Lysine	−	−	+	−
Ornithine	−	+	+	−
Urease	−	−	+	−
Phenylalanine Deamination	+	−	−	−
Nitrate reduction	−	−	+	−
H2S production	−	−	−	+
Citrate	+	−	+	+
Voges Proskauer	−	−	−	−
Methyl red	−	−	−	−
Indole	−	−	−	−
**Carbohydrate utilization test**				
Malonate utilization	+	+	+	−
Esculin hydrolysis	−	−	+	+
Arabinose	+	−	−	−
Xylose	+	−	−	−
Adonitol	−	−	−	−
Rhamnose	−	−	−	−
Cellobiose	−	−	−	−
Melibiose	−	−	−	−
Saccharose	−	−	−	−
Raffinose	−	−	−	+
Trehalose	−	−	−	+
Glucose	+	−	−	+
Lactose	−	−	−	+
Oxidase	+	−	+	−
Catalase	+	+	+	+

‘+/−’ indicates positive/negative response.

### PCR amplification of aromatic ring hydroxylating dioxygenase gene

Aerobic degradation of biphenyl is initiated by ARHD that converts biphenyl to 2,3-dihydroxy-1-phenylcyclohexa-4,6-diene (dihydrodiol compound). This enzyme has been reported to incorporate two hydroxyl groups on the aromatic ring, resulting in the opening of the aromatic ring (Furukawa et al., 2004; Kweon et al., 2008; Iwai et al., 2011). Therefore, in order to check the presence of the most crucial ARHD gene, PCR amplification was done. PCR amplification confirmed the presence of ARHD gene with an amplified product size of approximately 157, 199, 161, and 202 bp in MAPB-2, MAPB-6, MAPB-9, and MAPB-27, respectively. Sequencing and Blastn analysis of the amplified products confirmed the presence of dioxygenase gene in all the selected isolates. In this study, ARHD gene has been amplified which codes for initial enzyme for biphenyl degradation.

### Optimization of growth parameter for polychlorinated biphenyl-degrading bacterial isolates

**FIGURE 2:**
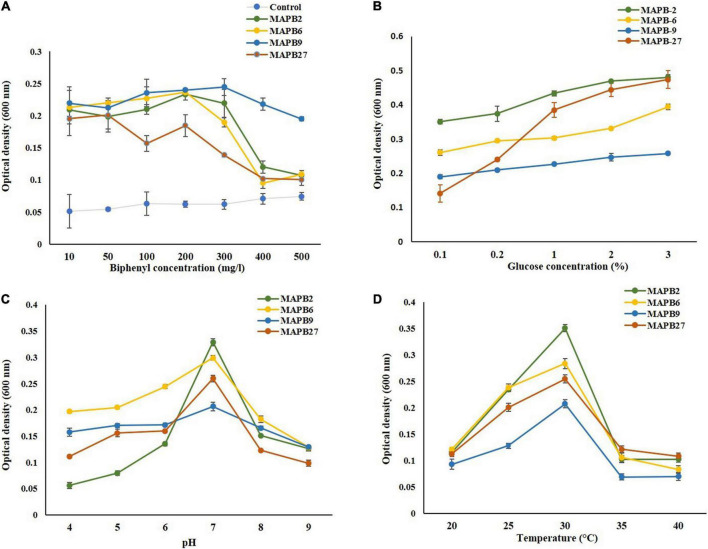
Optimization of the growth parameter for potential PCB degrading bacterial isolates grown in minimal medium in 48 h **(A)** biphenyl concentration range (10–500 mg/l) **(B)** glucose conc. (0.1–3%) with 200 mg/l biphenyl **(C)** pH (4-9) and **(D)** temperature (20–40°C).

Before conducting the biodegradation study, it is crucial to optimize the growth parameters for efficient PCB degradation. Various studies have been conducted on optimizing growth conditions to accomplish enhanced biodegradation efficiency (Khanpour-Alikelayeh et al., 2020). Several environmental factors such as the concentration of contaminant, presence of inducers, pH, and temperature, can alter bacterial growth that in turn influences biodegradation efficiency. The optimum concentration of biphenyl for bacterial growth varied for different isolates showing best growth by MAPB-2, MAPB-6, and MAPB-9 at 200 mg/l concentrations and 50 mg/l by MAPB-27 (Figure 2A). The differences in growth pattern of individual bacterial isolates with respect to different concentrations of biphenyl may be due to their different adaptive mechanisms (Chavez et al., 2006). It has been reported that biphenyl degradation ability gets stimulated *via* induction of the biphenyl catabolic enzymes. It has been reported that the addition of co-metabolite, such as glucose, provides an alternative nutrient and hence results in an increase in growth during PCB biodegradation, further resulting in enhanced degradation of PCB. The addition of glucose as co-metabolite improved the PCB degrading property of the bacterial isolates *via* growth stimulation. In addition, the best growth was observed at glucose conc. 3% in MM supplemented with 200 mg/l biphenyl for all the selected bacterial isolates (Figure 2B). Nevertheless, it was also observed that the rate of biodegradation decreases with a high amount of co-substrate, as a co-substrate is more rapidly utilized than PCB by bacteria (Bidlan and Manonmani, 2002). Therefore, 0.2% glucose conc. was selected for the further experiments, as it also supported growth of these isolates in low glucose conc. in the presence of 200 mg/l biphenyl. The optimum pH for PCB degradation was found to be 7 for all the isolates (Figure 2C) which tallies with previous studies reporting near-neutral pH as the most favorable for the bacterial degradation of chlorinated pollutant such as PCB (Fang et al., 2010). The reduced growth at acidic or alkaline pH strongly infers that bacterial growth is greatly influenced by a small alteration in the pH of the medium (Khopade et al., 2012). As observed for the pH, a same pattern was detected for the optimization of temperature as well. The temperature range of 20^–^40°C was optimal as shown in Figure 2D. Bacterial growth was severely suppressed at temperature 20, 35, and 40°C, while 25 and 30°C was found to be the optimum for growth. From these optimization studies, it could infer that variation in the growth parameters directly affects the growth of the isolates and indirectly the biodegradation rate. The overall result indicated that optimization of pH and temperature aids in the promoting bacterial growth in MM with 200 mg/l biphenyl concentration and 0.2%, glucose as a co-substrate. Therefore, the growth parameters of pH 7.0 and temperature 30°C were selected for the biodegradation studies of biphenyl and PCB-77.

### RNA extraction and reverse transcription polymerase chain reaction

**FIGURE 3:**
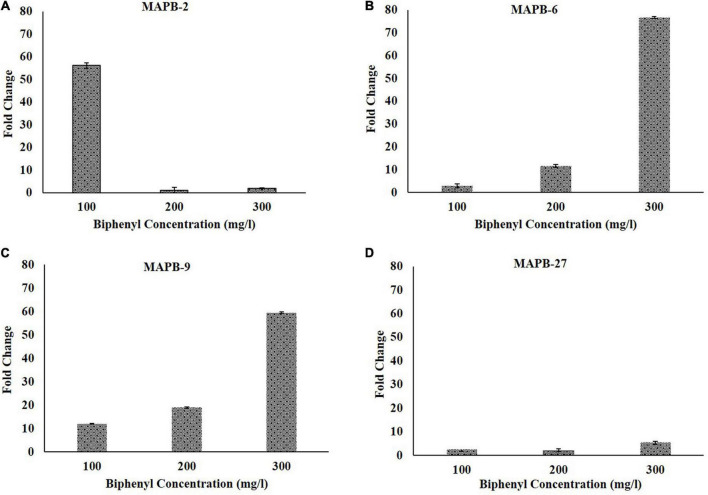
Gene expression of ARHD with increasing concentration of biphenyl at 100, 200, and 300 mg/l. **(A)** MAPB-2, **(B)** MAPB-6, **(C)** MAPB-9, and **(D)** MAPB-27.

The relative expression level of the ARHD gene in bacterial cultures grown with different concentrations (100, 200 and 300 mg/l of biphenyls) was compared with that of controls. In case of MAPB-2, an increase in biphenyl concentrations upregulated the ARHD gene expression by 56.1 and 1.4 in cultures treated with 100 mg/l and 300 mg/l biphenyls, respectively. However, the level of ARHD expression increased with increase in concentration of biphenyls in other strains. The increase in biphenyl concentrations to 100, 200, and 300 mg/l upregulated the ARHD gene expression to 2.8, 11.6, and 76.6 folds in MAPB-6; 12, 18.8, and 59.5 folds in MAPB-9 and 2.6, 2, and 5.5 folds in MAPB-27, respectively (Figure 3). The upregulation in MAPB-2, MAPB-6, MAPB-9, and MAPB-27 might be attributed to stress induced by biphenyl at 100, 200 and 300 mg/l to initiate the biphenyl degradation pathway through increased expression of ARHD (Chakraborty and Das, 2016).

### Biodegradation assay with biphenyl and PCB-77

#### Biodegradation assay with biphenyl

**FIGURE 4:**
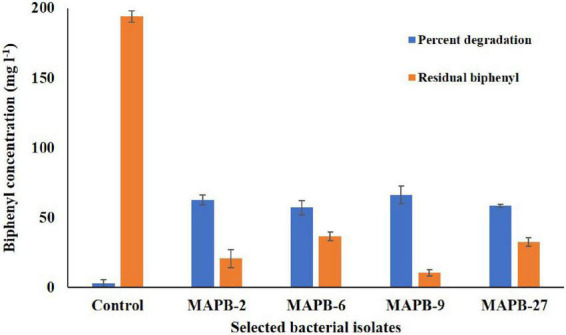
GC-MS/MS study for selected bacterial isolates grown in minimal media supplemented with 200 mg/l biphenyl at 30°C for 48 h, 150 rpm.

**FIGURE 5:**
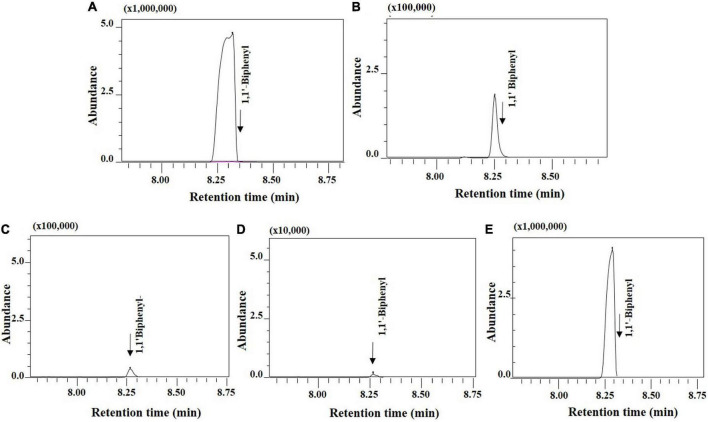
Enhanced percentage of biphenyl degradation (200 mg/l) kept at 30°C for 48 h with optimized parameters **(A)** control without inoculum **(B)** *P. aeruginosa* MAPB-2 showing 67.5% **(C)** *P. plecoglossicida* MAPB-6 showing 97.1% **(D)** *B. anthropi* MAPB-9 showing 100% **(E)** *P. megaterium* MAPB-27 showing 53.3% degradation.

To measure the efficiency of biphenyl and PCB degradation, all four selected bacterial isolates were subjected to GC-MS/MS analysis for percentage degradation of the pollutants. 200 mg/l biphenyl was used in the growth medium, as a sole carbon source. The percentage of biphenyl degradation was achieved up to 66.15, 62.06, 57.02, and 56.55% by *B. anthropi* MAPB-9, *P. aeruginosa* MAPB-2, *P. plecoglossicida* MAPB-6, and *P. megaterium* MAPB-27, respectively (Figure 4). However, the biphenyl degradation efficiency increased with the addition of co-substrate glucose (0.2%) to the culture medium showing a percentage degradation of 100, 97.1, 67.5, and 53.3% by MAPB-9, MAPB-6, MAPB-2, and MAPB-27, respectively (Figure 5) in 48 h. Thus, the present study revealed that each isolate behaved differently with respect to its biphenyl degrading potential when used as the sole source or co-metabolically with glucose. Our results are similar to previous studies which indicated the PCB degrading ability of different bacteria, such as *Mycobacterium* sp. PYR-1 degrades 98% biphenyl (80 mg/l) in 72 h (Moody et al., 2002), *Dyella ginsengisoli* LA-4 could degrade 95% biphenyl (100 mg/l) in 36 h (Li et al., 2009), *Achromobacter* sp. BP3 could be 100% biphenyl (50 mg/l) in 24 h (Hong et al., 2009). However, given studies did not test the degradation property in presence of co-metabolite. Similar study reported by Murinova et al. (2014), highlighted that the addition of glucose as co-metabolite enhanced bacterial growth and PCB biodegradation. Therefore, it appears that the efficiency and amount of PCB degradation depends not only on the type and concentration of the carbon source/inducer but also accustomed by the genetic setup of the bacterial isolate (Murinova et al., 2014).

##### Analysis of biphenyl metabolites

Biphenyl metabolites were extracted and analyzed by GC-MS/MS after the derivatization with BSTFA-TCMS. Benzoic acid (*m/z* = 194) was identified as one of the major metabolites during the biphenyl degradation by all the selected isolates. However, 3-hydroxybenzoic acid was identified in MAPB-2 while 2,3 dihydroxybenzoic acid was identified in MAPB-9 and MAPB-27. It is reported that aromatic ring of biphenyl is cleaved by enzyme dioxygenases and convert it into 2,3-dihydroxybiphenyl (2,3-DHB). Further, another BphC dioxygenase convert 2,3-DHB to 2-hydroxy-6-oxo-6-phenylhexa-2,4-dienoic acid (HOPDA). It is further cleaved into benzoic acid and 2-hydroxypenta 2,4-dienoic acid. Benzoic acid is considered to be a dead-end metabolite product during biphenyl degradation (Pieper and Seeger, 2008; Murinova et al., 2014). The identification of benzoic acid in the extract confirms that degradation of biphenyl is taking place in all the selected bacterial isolates. In addition, the identification of the hydroxylated benzoic acid (hydroxybenzoic in MAPB-2 and 2,3-hydroxybenzoic acid in MAPB-9, MAPB-27) in the extracts further reveals that benzoic acid degradation *via* protocatechuate by meta cleavage pathway follows upper biphenyl degradation pathway and channeled into central metabolism. Benzoic acid catabolic pathways such as the β-ketoadipate pathway are relatively widespread in the soil environment (Leewis et al., 2016).

#### Biodegradation assay with PCB-77

**FIGURE 6:**
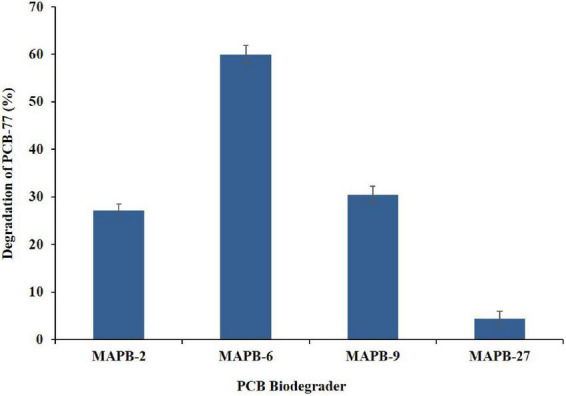
Percentage degradation of PCB 77 (50 mg/l) by MAPB-2, MAPB-6, MAPB-9, and MAPB-27 kept at 30°C for 7 d.

Biphenyl with more than two chlorine per molecule is mostly considered to be recalcitrant and is usually biodegraded *via* co-metabolism (Kang, 2014). PCB-77 consists of four chlorine molecules at *meta* and *para* positions. It is abundant within the food chain due to its extensive application in commercial blends such as Aroclor (Wang et al., 2016). Therefore, it is very important to explore the potential degrader for the degradation of such coplanar PCB. MAPB-2, MAPB-6, MAPB-9, and MAPB-27 were evaluated for degradation of PCB-77 in the presence of biphenyl as an inducer. Based on the data of GC-MS/MS analysis, all the isolates degraded PCB-77 (50 mg/l) in 7 d, though with varied efficiency. MAPB-6 showed the highest degradation of PCB-77 (59.89%) followed by MAPB-9 (30.49%), MAPB-2 (27.19%), and MAPB-27 (4.43%) as depicted in Figure 6. There are reports that, in the absence of an inducer, potent PCB-degrading bacteria such as *Rhodococcus* sp. RHA1 (0%), *P. pseudoalcaligenes* KF707 (0%) and *Burkholderia xenovorans* LB400 (6%) exhibited no or very low degradation of PCB-77 (Sakai et al., 2005). To our knowledge, this is the first study to report that *P. aeruginosa, P*. *plecoglossicida, B. anthropi*, and *P. megaterium* are capable of degrading PCB-77 with reasonable efficiency.

##### Analysis of PCB-77 metabolites

There is very less information related to PCB-77 metabolite degradation. However, benzoic acid and 3,3′,4 trichloro benzoic acid were detected in MAPB-2 at R_*t*_ 4.96 and 27.815 min, respectively. 3,4- Dihydroxybenzoic acid, 3TMS, and Dihydroxyacetophenone, 2TMS at 38.98 and 38.256 min in MAPB-6; 3,4 Dihydroxybenzoic acid, 3TMS at 41.165 min in MAPB-9, while 3,4 Dihydroxybenzoic acid, 3TMS and Dihydroxyacetophenone, 2TMS at 6.62 and 37.85 min in MAPB-27 were detected. PCBs undergoes dehalogenation to lower chlorinated biphenyl and further to chlorobenzoic acid metabolite. These chlorobiphenyl is further converted into hydroxybenzoic acid and dihydroxybenzoic acid (Zhai et al., 2010; Plotnikova et al., 2012). Bedard et al. (1987), have reported chlorinated benzoic acid and chlorinated acetophenone intermediate metabolites from oxidation of PCB congeners. However, it is not clear whether these metabolites arise from common or independent metabolic pathway.

### Biosurfactant production and characterization

#### Cetyltrimethylammonium bromide agar plate assay for screening of the biosurfactant

Bacterial biosurfactant-producing property is one the most important feature in biodegradation as biosurfactant enhances PCB availability from the contaminated soil, which in turn increases the biodegradation rates (Lászlová et al., 2018). CTAB-methylene blue agar plate method is a semi-quantitative screening assay for determining the production of glycolipid biosurfactant. The anionic surfactant produced by the bacteria interacts with the cationic surfactant CTAB, resulting in a dark blue insoluble ion pair with CTAB and methylene. Blue halo around the bacterial colonies, indicated that the selected isolates were capable of producing biosurfactants (Walter et al., 2010).

#### Emulsification index and drop collapse assay of biosurfactant

The efficacy of biosurfactants produced by MAPB-2, MAPB-6, MAPB-9, and MAPB-27 was tested against petroleum oil. *P. aeruginosa* MAPB-2 and *P. Plecoglossicida* MAPB-6 were found to exhibit the emulsifying activity of 66 and 57%, respectively. However, *P. megaterium* MAPB-27 showed the least emulsification at 44%. In addition, all isolates showed a positive response to the drop collapse assay, as the supernatant led to the expansion of the water by reducing interfacial tension, in turn increasing the drop size.

#### HPTLC and ATR-FTIR characterization of the biosurfactants

**FIGURE 7:**
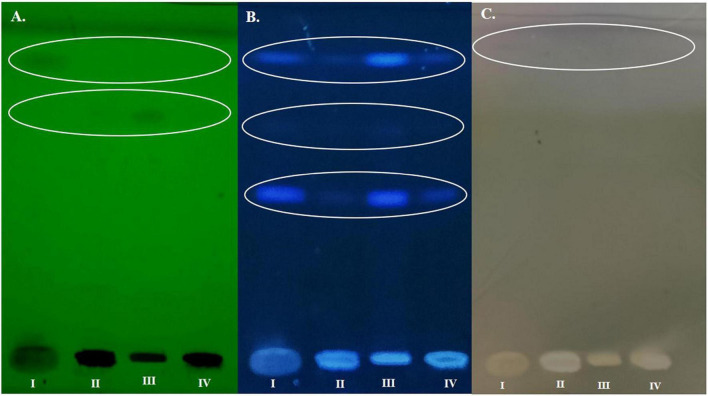
HPTLC based characterization of the biosurfactant produced by MAPB-2 (I) MAPB-9 (II) MAPB-6 (III) and MAPB-27 (IV) under **(A)** 254 nm, **(B)** 366 nm, and **(C)** derivatized with anisaldehyde.

The biosurfactant consists of a hydrophobic moiety that includes fatty acid, while the hydrophilic moiety can be carbohydrate, phosphate, carboxylic acid, alcohol and amino acid. Therefore, for further characterization of the biosurfactant, the extracted biosurfactant was analyzed by HPTLC and ATR-FTIR spectroscopy providing information on functional groups, bond, and molecular nature (Aparna et al., 2012; Ibrahim, 2018). In HPTLC analysis under short UV (254 nm) two bands at R_*f*_ 0.91 and 0.72 (as black spots) were observed, while at long UV (366 nm) in each sample (I, II, III, and IV) blue fluorescent bands were observed with R_*f*_ value of 0.54, 0.72 and 0.91 in all the four isolates. The HPTLC plate was derivatized with anisaldehyde reagent and it showed a pinkish black band with R_*f*_ value of 0.91 in MAPB-2 and MAPB-6 (Figure 7). The bands with an R_*f*_ value of 0.91 indicated the presence of carbohydrate moiety with p-anisaldehyde.

**FIGURE 8:**
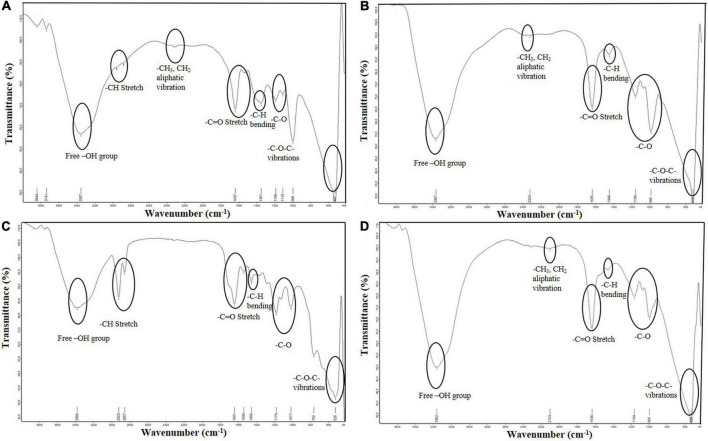
ATR-FTIR-based structural characterization of the biosurfactant produced **(A)** MAPB-2 **(B)** MAPB-6 **(C)** MAPB-9, and **(D)** MAPB-27.

The FTIR spectra showed broad and significant peaks at 3357, 3382, 3367, and 3362 cm^–1^ corresponding to -OH stretching of the glycolipid in MAPB-2, MAPB-6, MAPB-9, and MAPB-27, respectively, while peaks at 2900–2800 cm^–1^ represented aliphatic -CH_3_ and -CH_2_ vibrations. The major peaks found to be present at 1637, 1641, 1635, and 1636 cm^–1^ in MAPB-2, MAPB-6, MAPB-9, and MAPB-27, were due to -C = O (ester group) of the lipids, respectively. The vibration at 1351, 1445, 1448, and 1440 cm^–1^ represented -C-H group bending, while peaks at 527, 526, 518, and 622 cm^–1^ corresponded to stretching vibrations of glycosidic bonds (Figure 8). These data suggest that the biosurfactant belongs to the glycolipid class. *Pseudomonas* and *Bacillus* are the most studied genera for biosurfactant production (Gudina et al., 2015) with examples such as *Pseudomonas* BSB1 and BSB2, *Burkholderia* sp. WYAT7 (Ashitha et al., 2020), *Pseudomonas aeruginosa* NGB4 (Bhawsar and Singh, 2016). Similar band patterns have been reported in the above species with wavenumber being used to determine the chemical nature of the biosurfactants.

#### GC-MS/MS analysis and characterization of the biosurfactant

The GC-MS/MS analysis identified different lipids/fatty acids with biosurfactant properties. GC-MS/MS analysis and characterization of identified biosurfactants from MAPB-2, MAPB-6, MAPB-9, and MAPB-27 are shown in Table 4. The main components of the fatty acid found in the four strains were the ascorbic acid analogs, L-(+)-Ascorbic acid 2,6-dihexadecanoic acid, Dodecane, 2,4-Di-tert-butylphenol, n-Nonadecanol-1, 1,2-benzenedicarboxylic acid ester and Tetratetracontane. However, octadecanoic acid (Stearic acid; C18:0) was identified in MAPB-2, MAPB-6, and MAPB-27. Octadecanoic acid is a surface-active agent and was found to be one of the major components derived from fatty acids produced by *Pseudomonas*, which has excellent surfactant properties (Klein et al., 2013). Furthermore, 2-Octenoic acid, cis-, 10-Undecenoic acid, and methyl ester were also found as the fatty acid component of the biosurfactant produced by *Pseudomonas* MAPB-2 and MAPB-6 (Sakthipriya et al., 2015). 1-Hexadecanol and Hexadecen-1-ol, trans- 9-, 1-Heneicosanol, 1-Dodecanol, and 1-Octanol, were found to be fatty alcohol component of the nonionic biosurfactant identified in MAPB-2. 1-Hexadecanol, n-Tridecan-1-ol, n-Tridecan-1-ol, and n-Tridecan-1-ol were identified as the main fatty alcohol component of biosurfactant produced by MAPB-2. Fatty acid component decanoic acid, octanoic, heptadecane, and fatty alcohol decanol were found to be specific to the biosurfactant extract of MAPB-6. While 2-hexanol, 2- methyl-, n-Tridecan-1-ol, n-Nonadecanol-1, Tridecanol, 2-ethyl-2- methyl-, n- Tetracosanol-, trans-2-Undecen-1-ol were found to be the main fatty alcohol found in MAPB-9. 4-hydroxy- 2-Pentenoic acid, is an α-alkyl hydroxy fatty acid identified in biosurfactant produced by MAPB-9. Therefore, considering biosurfactants MAPB-2 and MAPB-6 were found to be good biosurfactant producers followed by MAPB-9, and MAPB-27. The improved PCB degradation (Aroclor 1248) spiked soil by bacterial surfactant rhamnolipid was demonstrated earlier by Cho et al. (2004). Thus, the detailed characterization done with different analytical techniques such as HPTLC, ATR-FTIR, and GC-MS/MS have highlighted the presence and nature of the biosurfactant produced by the selected isolates.

**TABLE 4:** GC-MS/MS analysis and characterization of identified compound from biosurfactant.

Rt	Name of the Compound	Molecular weight	Chemical formula	Isolates MAPB			
				2	6	9	27
5.31	2-Hexanol, 2-methyl-	116.2	C_7_H_16_O	✕	✕	✓	✕
8.05	Decanoic acid, hexyl ester	256.4	C_16_H_32_O_2_	✕	✓	✕	✕
9.41	1-Nonen-4-ol	142.2	C_9_H_18_O	✕	✕	✓	✕
10.19	Octanoic acid, octyl ester	256.4	C_16_H_32_O_2_	✕	✓	✕	✕
17.02	Dodecane	170.3	C_12_H_26_	✓	✓	✓	✓
17.57	2-Pentenoic acid, 4-hydroxy-	116.1	C_5_H_8_O_3_	✕	✕	✓	✕
19.10	1-Decanol, 2-hexyl-	242.4	C_16_H_34_O	✕	✓	✕	✕
20.34	Dodecane, 2,6,11-trimethyl-	212.4	C_15_H_32_	✓	✕	✕	✕
20.36	Tetradecane	198.3	C_14_H_30_	✕	✕	✓	✕
20.38	Heptadecane, 8-methyl-	254.4	C_18_H_38_	✕	✓	✕	✕
21.35	1-Undecanol	172.3	C_11_H_24_O	✕	✕	✓	✕
21.52	2,4-Di-tert-butylphenol	206.3	C_14_H_22_O	✓	✓	✓	✓
22.12	Hexadecane, 2,6,10,14-tetramethyl-	282.5	C_20_H_42_	✓	✓	✓	✕
22.12	Pentadecane, 3-methyl-	226.4	C_16_H_34_	✓	✕	✓	✕
22.33	1-Hexadecanol	242.4	C_16_H_34_O	✓	✕	✕	✓
22.34	n-Tridecan-1-ol	200.3	C_13_H_28_O	✕	✓	✓	✓
22.41	Eicosane	282.5	C_20_H_42_	✓	✓	✓	✓
23.62	Hexacosane	366.7	C_26_H_54_	✓	✕	✕	✕
23.67	Tetradecane, 5-methyl-	212.4	C_15_H_32_	✓	✓	✓	✕
23.68	Pentadecane, 8-hexyl-	296.5	C_21_H_44_	✕	✕	✓	✕
23.84	Heptadecane, 2-methyl-	254.4	C_18_H_38_	✓	✓	✓	✕
24.02	n-Nonadecanol-1	284.5	C_19_H_40_O	✓	✓	✓	✓
22.43	Octadecane, 5-methyl-	268.5	C_19_H_40_	✕	✓	✕	✓
24.58	1,2-Benzenedicarboxylic acid ester	278.3	C_16_H_22_O_4_	✓	✓	✓	✓
24.99	Hentriacontane	436.8	C_31_H_64_	✓	✕	✕	✕
25.84	trans-2-Undecen-1-ol	170.2	C_11_H_22_O	✓	✕	✓	✓
26.53	Nonadecane, 9-methyl-	282.5	C_20_H_42_	✕	✓	✕	✓
26.54	Heptadecane, 2,3-dimethyl-	268.5	C_19_H_40_	✓	✓	✓	✕
26.61	Tridecanol, 2-ethyl-2-methyl-	242.4	C_16_H_34_	✕	✕	✓	✕
26.62	Heneicosane, 5-methyl-	310.6	C_22_H_46_	✓	✓	✓	✕
26.75	Octadecanoic acid (Stearic acid)	284.4	C_18_H_36_O_2_	✓	✓	✕	✓
26.92	n-Tetracosanol-1	354.6	C_24_H_50_O	✕	✓	✓	✕
26.97	Heneicosane	296.5	C_21_H_44_	✓	✓	✓	✕
27.12	1-Heneicosanol	354.6	CH_3_(CH_2_)_2_	✓	✓	✕	✓
27.65	Hexadecen-1-ol, trans-9-	240.4	C_16_H_32_O	✓	✕	✕	✕
28.44	Tetratetracontane	619.2	C_44_H_90_	✕	✓	✓	✓
30.37	Tetracontane	563	C_40_H_82_	✓	✕	✕	✕
35.66	E-3-Pentadecen-2-ol	226.4	C_15_H_30_O	✕	✕	✓	✕

✕/✓ symbols indicate absence/presence of the compound.

## Conclusion

In conclusion, *P. aeruginosa* MAPB-2, *P. plecoglossicida* MAPB-6, *B. anthropi* MAPB-9, and *P. megaterium* MAPB-27 with high biphenyl tolerances and degradation efficiency were isolated from PCB-contaminated soil. Isolates MAPB-2, MAPB-6, MAPB-9, and MAPB-27 could tolerate up to 500 mg/l biphenyl. Optimized growth conditions led to 100% improved biphenyl biodegradation by MAPB-9 with 200 mg/l biphenyl, while 97.1 for MAPB-2 within 48 h, respectively. Aromatic ring cleavage dioxygenase gene was amplified and upregulated in biphenyl induced condition in all the isolated strains. The study is the first report of effective PCB-77 degradation with 59.89, 30.49, 27.19, and 4.43% degradation by MAPB-6, MAPB-9, MAPB-2, and MAPB-27, respectively, using biphenyl as inducers. In addition to biodegradation potential, the isolates were also found to be capable of producing biosurfactants. The biosurfactant-producing property of these isolates can be attributed to good PCB degradation. Thus, the results suggest that these bacteria might be potential candidates for bioremediation of environments contaminated with biphenyl/PCBs.

## Data availability statement

The datasets presented in this study can be found in online repositories. The names of the repository/repositories and accession number(s) can be found in the article/supplementary material.

## Author contributions

MS and PJ designed the project. AP designed the FTIR, HPTLC, and GC-MS/MS-based experiment and analyzed the respective data. MS conducted all the experiments. MS, AP, JP, JL, and PJ analyzed and interpreted the results and wrote the manuscript. All authors contributed to the finalization of the manuscript.

## Footnotes

1
http://www.ncbi.nlm.nih.gov/BLAST/
